# Computational study of ‘HUB’ microRNA in human cardiac diseases

**DOI:** 10.6026/97320630013017

**Published:** 2017-01-24

**Authors:** Remya Krishnan, Achuthsankar S. Nair, Pawan K. Dhar

**Affiliations:** 1 Department of Computational Biology and Bioinformatics, University of Kerala, Thiruvananthapuram, Kerala – 695 581;; 2 School of Biotechnology, Jawaharlal Nehru University, New Delhi 110067;

**Keywords:** disease network, dys-regulation, miRNAs, statistical analysis

## Abstract

MicroRNAs (miRNAs) are small non-coding RNAs ~22 nucleotides long that do not encode for proteins but have been reported to influence gene expression in normal and abnormal health conditions. Though a large body of scientific literature on miRNAs exists, their network level profile linking molecules with their corresponding phenotypes, is less explored. Here, we studied a network of 191 human miRNAs reported to play a role in 30 human cardiac diseases. Our aim was to study miRNA network properties like hubness and preferred associations, using data mining, network graph theory and statistical analysis. A total of 16 miRNAs were found to have a disease node connectivity of >5 edges (i.e., they were linked to more than 5 diseases) and were considered hubs in the miRNAcardiac disease network. Alternatively, when diseases were considered as hubs, >10 of miRNAs showed up on each ‘disease hub node’. Of all the miRNAs associated with diseases, 19 miRNAs (19/24= 79.1% of upregulated events) were found to be upregulated in atherosclerosis. The data suggest micro RNAs as early stage biological markers in cardiac conditions with potential towards microRNA based therapeutics.

## Background

MicroRNAs are non-coding RNAs that are transcribed but do not encode proteins. miRNAs have been reported to play a pivotal role in regulating key biological processes e.g., posttranscriptional modifications and translation processes. Ever since the discovery of miRNAs in 1993, they have been reported to influence a variety of human physiological conditions such as post transcriptional regulation of skeletal development [1], circadian rhythms [2], cancers [3-4], and neuro-degenerative diseases like Alzheimer’s [5], cardiac disorders [6], etc. MicroRNAs are robust and diverse in functions and are also predicted to control human ageing process [7]. A more recent study has revealed that cardiac microRNAs can indirectly regulate the function of other cardiac microRNAs ie, microRNAmediated microRNA regulation. A number of experimental as well as computational studies have supported the role of microRNA regulators in heart diseases like Myocardial Infarction, Cardiomyopathies, Atherosclerosis, Stenosis, etc.

Cardiovascular diseases (CVDs) are one of the leading causes of human deaths [8]. The global status report of the World Health Organization [9] reported an estimated 17.3 million people having died of CVD as of 2008. This number has been projected to increase to 23.3 million people dying of heart diseases alone by the year 2030. To understand a complex heart condition such as hypertrophic cardiomyopathy or atherosclerosis in terms of causes and treatment it‘s important to study the molecular inventory and explore the network organizes itself to produce a certain phenotype, in normal and diseased states. Using computational approach, an attempt was made to find an overall interaction pattern of experimentally validated microRNAs in heart conditions, based on previous reports associating microRNAs with clinical phenotypes. We were interested to find if microRNAs “hubs” existed in disease networks and, if yes, could they be used as biomarkers before / during the onset of disease conditions? Also, if micro RNA associations existed, could one use them as potential therapeutic agents to treat cardiac conditions?

In this study, a total of 28 different human heart diseases were identified from the Human MicroRNA Disease Database (HMDD: http://202.38.126.151/hmdd/mirna/md/) and mapped to 190 experimentally validated microRNAs associations.

## Discussion

**Figure 1:**
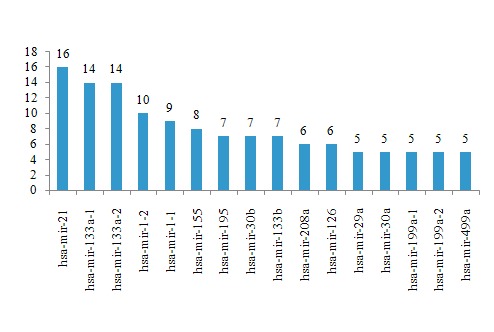
The frequency of microRNA- cardiac disease incidence

**Figure 2:**
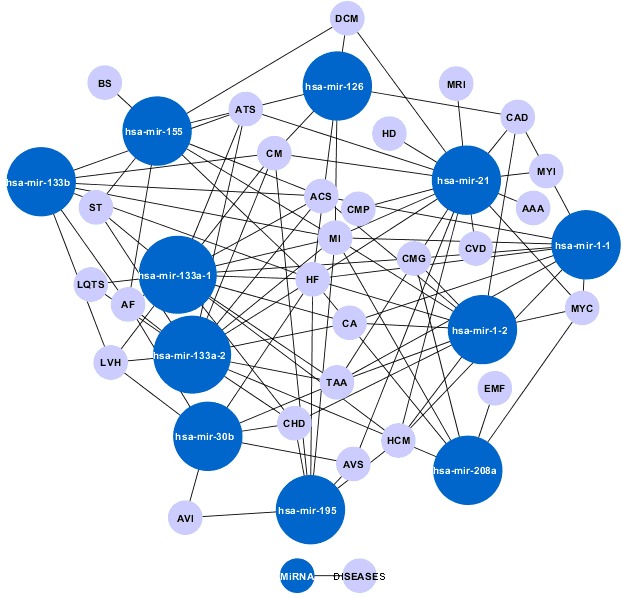
The microRNA-cardiac disease network. (nodes indicated in blue are hub micro RNAs and grey color nodes are diseases. All the blue nodes are hubs implicated in various cardiac diseases). The abbreviated names are read as follows: EMF- Endomyocardial Fibrosis; CMG- Cardiomegaly; CACardiac arrhythmiasis; MYC- Myocardium; CAD- Coronary Artery Disease; ATS- Atherosclerosis; CM- Cardiac myocytes; LQTS- Long QT syndrome; ACS- Acute Coronary Syndrome; HCM- Hypertrophic cardiomyopathy; ST- Stroke; TAA- Thoracic Aortic Aneurysm; MI- Myocardial Infarction; CHD- Congenital Heart Defects; HF- Heart Failure; BS- Behcet Syndrome; AF- Atrial Fibrillation; LVH- Left Ventricular Hypertrophy; AVS- Aortic Valve Stenosis; MYI- Myocardial Ischema; HD- Heart diseases; AAA- Abdominal Aortic Aneurysm; CMP- Cardiomyopathies; MRI- Myocardial Reperfusion Injury; CVD- Cardiovascular Disease

MicroRNAs that occurred most frequently in human cardiac diseases obtained from HMDD are shown in the Figure 1. From the data, several hub microRNAs with more than 5 disease associations were found. Based on the derived data, Figure 2 shows a bi-partite graph based representation of the microRNAcardiac disease network generated using Cytoscape.

In order to perform the Fisher’s two-tailed test, 191 microRNAs associations with 30 cardiac diseases were recorded as 388 disease incidences, as one microRNA was found to dysregulate more than one cardiac disease. The entire dataset comprised of 388 disease incidences, 224 microRNA upregulation events and 164 down-regulation events. MicroRNAs linked with cardiac diseases like atherosclerosis (P = 0.0001); coronary artery diseases (P = 0.0001); cardiomegaly (P=0.0002) and other statistically significant associations (Table 1) show distinct dysregulation pattern in a disease specific manner. Up to 19 miRNAs (19/24=79.1% of total upregulated events) were found to be up regulated in atherosclerosis. This emphasizes that the products of genes linked with a disordered associations, interact more with disordered molecules than with others. Hence during or before the onset of a cardiac disease significant number of microRNAs are attracted to interact with one another with distinct dysregulation patterns making the altered physiological state to act as hubs. Thus microRNAs can be used as biomarkers for identifying such states.

To understand the hidden patterns and trends in large-scale data, Knowledge discovery or data mining methods can be employed. Data mining uses several methods to identify the implicit information, one of which is Association rule mining (ARM). ARM employs algorithms such as Apriori [15], FP Growth [16], Tree Projection [17], etc. We have used the FP Growth algorithm for association rule mining in order to identify novel patterns of associations of all the microRNAs implicated in human cardiac diseases.Most algorithms used in ARM are derivatives of the Apriori algorithm. Since the Apriori algorithm has disadvantages in candidate set generation; the FP Growth algorithm was developed to overcome this problem. The FP Growth algorithm uses a partition-based technique and identifies frequent patterns by pattern fragment growth. SPMF (Sequential Pattern Mining Framework) generated 50 association rules and 26 frequent item sets from 191 microRNAs with a pre-defined threshold support and confidence of 0.2 and 0.6 respectively. Only the biologically significant validated and novel associations are listed in Table 2.

MicroRNAs are master regulators of gene functions. They can manifest in robust ways, hence being able to predict the nature of their behavior in a particular disease phenotype is a boon. Computational biology algorithms, techniques and bioinformatics tools have enhanced the significance of predictive science. In this study, predictive behavior of microRNAs in cardiac diseases has been concluded. This is a milestone in adding to the therapeutic potentials of microRNAs in terms of gene-based therapy. Signature expression patterns marks a flag post for where the experimentalists ought to look for. This study also proves the potential of miRNA genes to function as molecular markers, which can be considered as a pre-diagnostic method to predict the onset of a disease. However the assessment of the above can be done only by experimental validation.

Currently individual experiments are being carried out to probe microRNA expression analysis with implications in several diseases. Even though data integration and updating schema is still a challenge however the bigger challenge lies in identifying and predicting the patterns such as miRNA dysregulation states, chromosome location based co-expression patterns, network modules etc.

## Conclusion

Of 191 experimentally validated microRNAs, about 16 microRNAs were identified to act as "hubs" in the microRNAcardiac disease network. The visualization was done by treating the network as a bi-partite graph based on graph theory analysis. The graph revealed "hubs" in two contexts: 1) disease 2) microRNAs. With reference to Table 1, it is evident that diseases tend to act as “hubs” that attract microRNAs with specific dysregulation patterns. This is statistical proof that if for example: In Stroke 88.8% (8/9) microRNAs are upregulated leading to a collective phenotypic condition leading to stroke, the statistical significance of which was confirmed by Fisher Two tail test similarly up to 19 miRNAs (19/24=79.1% of total upregulated events) were found to be up regulated in atherosclerosis. There may be several other players involved however the presence of “hub microRNAs” like miR 133 a-1; miR 133 a-2 and miR 155 in the stroke and atherosclerosis condition in an upregulated state enables the disease phenotype itself to act as a hub attracting other microRNAs that are mostly upregulated thus proving that the products of genes linked with a disordered associations, interact more with disordered molecules than with others [18]. Our study therefore emphasizes that judicial use of computational analysis techniques for large scale biological data analysis would reveal novel biological insights towards therapeutics which may not be apparent by individual experimental analysis alone.

## Figures and Tables

**Table 1:** Disease specific dysregulation pattern

S. No.	Disease	% involvement	Up/Down regulation	p value
1	Cornonary artery disease	75 % (15/20)	UP	0.0001
2	Heart Failure	53 % (70/132)	UP	0.001
3	Hypertrophic cardiomyopathy	52.1 % (12/23)	UP	0.0009
4	Myocardial Infarction	52.7 % (19/36)	UP	0.026
5	Myocardial Ischema	71.4 % (5/7)	UP	0.001
6	Myocardial reperfusion injury	100 % (5/5)	UP	0.0001
7	Myocarditis	100% (2/2)	UP	0.024
8	Stroke	88.8% (8/9)	UP	0.0001
9	Thoracic aneurysm	60% (9/15)	DOWN	0.0006

The p values in the bold are extremely significant				

**Table 2:** Novel cluster associations of microRNAs

miRNA associations	Disease type	Dys-regulation pattern	Type
1-1, 1-2 => 133a-2	TAA, CMG, HCM	D, D, D	Validated [2], [4], [19], [20]
1-1,133a-2 =>1-2,133a-1	TAA, CMG, HCM	D, D, D, D	Validated [2], [4], [19], [20]
133 a-1, 133b => 133a-2	AF	D, D, D	Predicted
133a-1, 155 => 133a-2	LVH, HCM, DCM	D, D, D	Predicted
155 ==> 133a-1	DCM, HCM	D, D	Predicted
155 ==> 133a-1, 133a-2	LVH, HCM, DCM	D, D, D	Predicted
155 ==> 133a-2	DCM, HCM, LVH	D, D	Predicted

TAA = Thoracic Aortic Aneurysm, CMG = Cardiomegaly, AF = Atrial Fibrillation LVH = Left Ventricular Hypertrophy, HCM = Hypertrophic Cardiomegaly, DCM = Dilated Cardiomyopathy D = Downregulated
